# Emerging Healthcare Threat Candida auris: A Prevalence Study From a Rural Tertiary Referral Centre in Western India

**DOI:** 10.7759/cureus.72676

**Published:** 2024-10-30

**Authors:** Savita B Tajane, Satyajeet Pawar, Satish Patil

**Affiliations:** 1 Department of Microbiology, Krishna Institute of Medical Sciences, Krishna Vishwa Vidyapeeth (Deemed to be University), Karad, IND

**Keywords:** candida auris, candidiasis, drug-resistant fungi, urine, uti

## Abstract

Background

*Candida auris* has emerged as an important healthcare-associated pathogen that has high mortality rates. Additionally, this pathogen can cause nosocomial outbreaks. However, in comparison to the vast majority of the pathogenic species from the genus Candida, *C. auris* is difficult to treat and identify by using conventional therapeutic and diagnostic modalities. The exact prevalence of this pathogen is largely unclear from Indian healthcare setups. This study aimed to determine the prevalence of *C. auris* in a tertiary care academic hospital in western Maharashtra and to study the associated risk factors of patients with *C. auris* infection.

Methods

Candida isolates were identified using conventional methods, including the germ tube test and colony morphology assessment on HiChrom™ Candida differential agar (Hi Media, Thane, India). Species-level identification was performed with the VITEK 2 system (version 8.01, BioMérieux, Marcy-l'Étoile, France), and *Candida auris* was confirmed using Matrix-Assisted Laser Desorption Ionization-Time of Flight Mass Spectrometry (MALDI-TOF-MS). Patient records were retrieved from patient files and reviewed retrospectively for demographic variables, risk factors, clinical conditions, treatment, and outcome.

Results

*C. auris* was isolated from nine clinical specimens. The prevalence of *C. auris* was 9.4% among non-albicans Candida species (n=96), whereas the prevalence of *C. auris* among total Candida isolates (n=147) was noted to be 6.1%. Multiple hospitalizations, ICU stay, comorbidities like chronic kidney disease, diabetes mellitus and hypertension along with concomitant bacterial infection and broad-spectrum antimicrobial therapy were associated risk factors.

Conclusion

In this study, *C. auris* was most predominantly isolated from urine specimens. The urinary tract infection (UTI) should not be ignored as at times it may progress to disseminated infection, especially in patients with comorbidities. The current study underlines the importance of automated systems for identifying *C. auris*. Prompt identification will aid in treatment whereas the implementation of infection prevention and control measures will help in the containment of infection spread in a healthcare setup.

## Introduction

As evidenced by increased scientific publications, candidiasis has gained the attention of both scientific and medical fraternities in recent years owing to its high frequency of occurrence and varied and wide range of clinical manifestations. Although aspects of Candida infections, once largely unknown have been elicited, many are yet to be unfolded.

With advancements in diagnostic modalities including radio-imaging and laboratory medicine various new clinical manifestations and species of Candida, hitherto unknown are increasingly recognized [1].

*Candida auris* is one such species that has gained attention in recent years and poses a significant problem to microbiologists and clinicians [2]. This novel mycotic pathogen was discovered in 2009 by Satoh et al from the ear canal of a Japanese patient and was named accordingly ('auris' in Latin means ear) [3]. However, after the availability of specific diagnostic methods such as MALDI-TOF mass spectrometry, *C. auris* was reported to be identified from retrospective strain collection maintained since the year 1996. Other specific diagnostic techniques include VITEK 2 (BioMérieux, Marcy-l'Étoile, France), real-time polymerase chain reaction (PCR), and next-generation sequencing (NGS) for precise identification. Specific identification systems are crucial to avoid misidentifying *C. auris* as other Candida species, which can lead to ineffective treatments [4].

 Although *C. auris* was for the first time isolated from the ear canal, it is most predominantly isolated from bloodstream infections (BSI). It has every potential to disseminate to internal organs through BSI and is responsible for high mortality [5].

Its ability to disseminate in hospital environments, its capability to colonize human skin, hospital environmental surfaces, and medical devices, and its rapid development of resistance to antifungal drugs make *C. auris* a worrisome pathogen [6]. *C. auris* is reported from nosocomial outbreaks across almost all continents of the world [6].

However, the exact prevalence of *C. auris* is largely unknown in Indian healthcare setups. The present research was conducted with an aim to determine the prevalence of *C. auris* in a tertiary care academic hospital in Western Maharashtra, India, and to study the associated risk factors of *C. auris* infection.

## Materials and methods

This study was conducted at the Department of Microbiology, Krishna Institute of Sciences (KIMS), Karad, Maharashtra for a period of 10 months (May 2023 to February 2024). Krishna Hospital is an 1125-bed super specialty hospital that caters a quality care to rural masses. The study protocol received approval from the Institutional Ethics Committee (452/2022-2023).

This descriptive cross-sectional study included 2014 clinical specimens received during the study period and identified 147 Candida isolates, with *Candida auris* confirmed in nine cases, which were the focus of further study. The laboratory workup for identifying Candida species began with conventional methodologies. All isolates were first evaluated using the germ tube test, where positive results indicated a presumptive identification of *C. albicans*. Isolates that tested negative for the germ tube were then cultured on chromogenic media (HiChrom™ Candida differential agar, Hi Media, Thane, India), which aids in differentiating species based on colony color and morphology, and then incubated at 37°C for 48 hours, with identification based on colony color and morphology. Following this initial identification, all isolates underwent species-level identification using the automated identification system VITEK 2 (version 8.01, BioMérieux). Additionally, *Candida auris* identified by VITEK 2 was confirmed using Matrix-Assisted Laser Desorption Ionization-Time of Flight Mass Spectrometry (MALDI-TOF-MS) technology [7]. The relevant clinical record of the patient was retrieved from the patient’s case sheet and reviewed retrospectively for demographic variables, risk factors, clinical conditions, treatment, and outcome. The data was entered in Microsoft Excel (Microsoft Corporation, Redmond, USA) and analyzed using appropriate descriptive statistics, figures, tables, and graphs.

## Results

**Figure 1:**
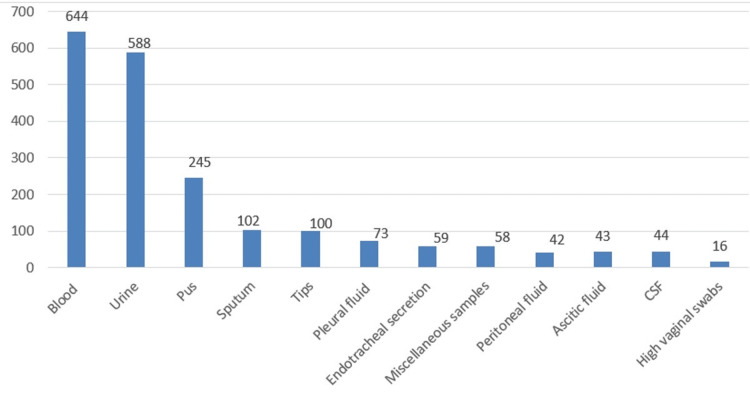
The details of clinical specimens in the study CSF: cerebrospinal fluid

During the study, a total of 2014 clinical specimens were received in the departmental laboratory from different outpatient departments (OPD), wards, and critical care facilities like intensive care unit (ICU), neonatal intensive care unit (NICU), and pediatric intensive care unit (PICU). The details of clinical specimens in the study are shown in Figure 1.

Out of 2014 clinical specimens received for culture and sensitivity, a total of 1006 (49.9%) were culture positive (showed growth). The rates of isolation of bacterial and fungal pathogens were 85.38% (n=859) and 14.61% (n=147) respectively. All fungal pathogens were Candida species.

These 147 Candida isolates were initially screened using the germ tube test and Hicrome™ Candida Agar, with species-level identification performed using the VITEK-2 system. Of these, nine *Candida auris* isolates were confirmed by MALDI-TOF. All results from VITEK-2 and MALDI-TOF for *Candida auris* were consistent.

The distribution of Candida isolates from various clinical specimens is shown in Table 1. The majority of Candida species were isolated from urine specimens (102, 69.4%). A total of 16 (10.9%) isolates were obtained from blood cultures whereas four (2.8%) isolates were from peritoneal fluid.

**Table 1:** Clinical specimen-wise distribution of Candida isolates Tips/lines included central venous pressure line, peripherally inserted central catheter etc.; %: percentage

Clinical specimens	Candida isolate (%)
Urine	102 (69.4)
Blood	16 (10.9)
Sputum	10 (6.8)
Tips/line	10 (6.8)
Peritoneal fluid	04 (2.7)
Endotracheal secretion	02 (1.4)
Pus	02 (1.4)
High vaginal swab	01 (0.7)
Total	147

**Figure 2:**
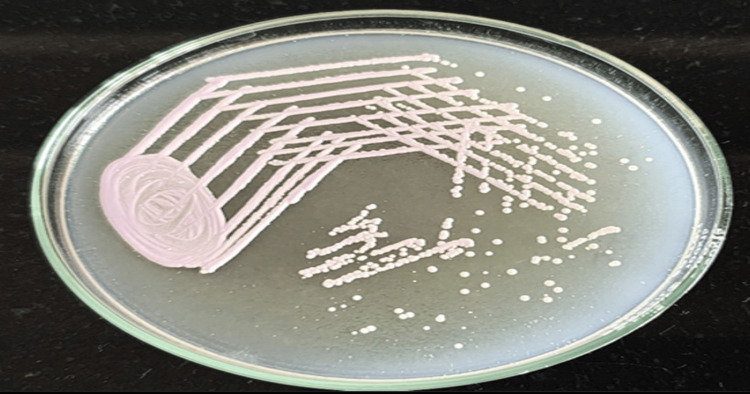
Candida auris colonies on HiChrome Candida differential agar

Non-albicans Candida species were identified on HiCrome™ Candida differential agar by their distinct colony colors, such as *Candida auris* (white or pink), as shown in Figure 2.

**Figure 3:**
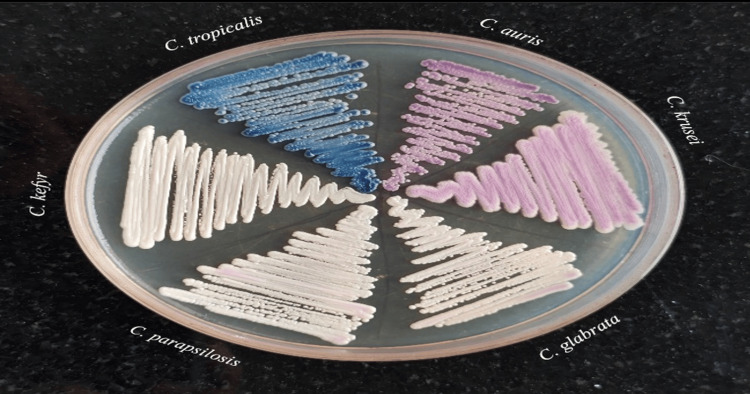
Non-albicans Candida species colonies on HiChrome Candida differential agar *C. auris*: *candida auris*, *C. tropicalis*: *candida tropicalis*, *C. krusei*: *candida krusei*, *C. glabrata*: *candida glabrata*, *C. parapsilosis*: *candida parapsilosis*, *C. kefyr*: *candida kefyr*

Other non-albicans Candida (NAC) species were identified, such as *C. tropicalis* (blue to purple), *C. krusei* (large, fuzzy pink), *C. glabrata* (pale pink to white), and *C. parapsilosis* (creamy white), *C. kefyr* (white) as shown in Figure 3. This helps in species differentiation.

**Figure 4:**
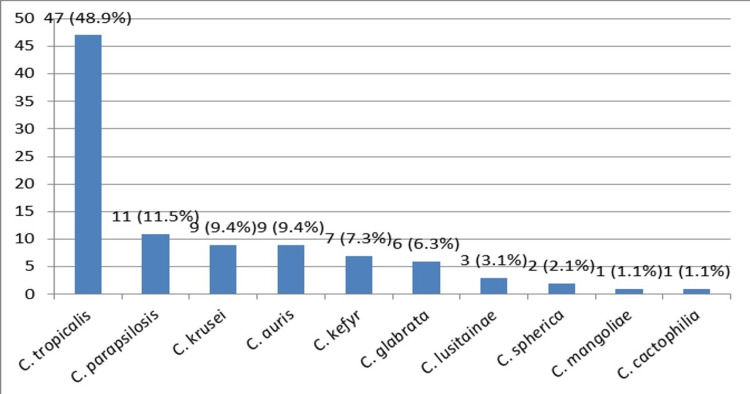
The species wise distribution of non-albicans Candida species *C. tropicalis*: *candida tropicalis*; *C. parapsilosis*: *candida parapsilosis*; *C. krusei*: *candida krusei*;* C. auris*: *candida auris*; *C. kefyr*: *candida kefyr*; *C*. *glabrata*: *candida glabrata*; *C. lusitainae* :*candida lusitainae*; *C. spherica*: *Candida spherica*; *C. mangoliae*: *candida mangoliae*; *C. cactophilia*: *candida cactophilia*

The isolation of NAC species from various clinical specimens is shown in Figure 4. *C. tropicalis* (48.9%) was the commonest NAC species, and *C. auris* was isolated from nine (9.4%) cases. The prevalence of *C. auris* in the current study was 9.4% among the NAC species (n=96).

Table 2 summarises the clinical specimen-wise distribution of Candida species. The predominance of NAC species was noted. NAC species were isolated from 96 (65.3%) clinical specimens, whereas *C. albicans* (34.7%) were isolated from 51 specimens. Out of a total of nine isolates of *C. auris*, five were from urine culture, two were from blood culture, whereas one isolate each was from SRC (self-retaining catheter) tip and high vaginal swab (HVS). These included two isolates of *C. auris* isolated from a single patient (urine and SRC tip). This may be a consequence of biofilm formation on SRC and the pathogen gaining entry into the urinary tract and further causing infection. All isolates from sputum and pus were *C. albicans*. Endotracheal secretion showed growth of *C. krusei* only. *C. kefyr* was obtained only from urine specimens. Prevalence of *C. auris* among Candida species (n=147) was noted to be 6.1%. 

**Table 2:** Clinical specimen wise distribution of Candida species. spp : species;c*C. tropicalis*: *candida tropicalis*; *C. parapsilosis*: *candida parapsilosis*; *C. krusei*: *candida krusei*; *C. auris*: *candida auris*; *C. kefyr*: *candida kefyr*; *C. glabrata*: *candida glabrata*; *C. lusitainae*: *candida lusitainae*; *C. spherica*: *Candida spherica*; *C. mangoliae*: *candida mangoliae*; *C. cactophilia*: *candida cactophilia*; HVS: high vaginal swab; %: percentage

Candida spp.	Urine (%)	Blood (%)	Sputum (%)	Tips (%)	Peritoneal fluid (%)	Miscellaneous specimens (%)	Endotracheal secretion (%)	Pus (%)	HVS	Total (%)
C. albicans	35 (34.3)	04 (25)	10 (100)	-	-	-	-	02 (100)	-	51 (34.7)
C. tropicalis	36 (35.3)	05 (31.3)	-	03 (42.9)	02 (50)	01 (33.3)	-	-	-	47 (31.9)
C. parapsilosis	04 (3.9)	03 (18.8)	-	02 (28.6)	-	02 (66.6)	-	-	-	11 (7.5)
C. krusei	04 (3.9)	01 (6.3)	-		02 (50)	-	02 (100)	-	-	09 (6.1)
C. kefyr	07 (6.9)	-	-	-	-	-	-	-	-	07 (4.8)
C. glabrata	05 (4.9)	-	-	01 (14.3)	-	-	-	-	-	06 (4.1)
C. auris	05 (4.9)	02 (12.5)	-	01 (14.3)	-	-	-	-	01 (100)	09 (6.1)
C. lusitaniae	03 (2.9)	-	-	-	-	-	-	-	-	03 (2.1)
C. spherica	01 (0.9)	01 (6.3)	-	-	-	-	-	-	-	02 (1.4)
C. mangoliae	01 (0.9)	-	-	-	-	-	-	-	-	01 (0.7)
C. cactophila	01 (0.9)	-	-	-	-	-	-	-	-	01 (0.7)
Total	102	16	10	07	04	03	02	02	01	147

When risk factors were analyzed for Candida infection, it was observed that diabetes mellitus (DM), pregnancy, female gender, and catheterization were major risk factors for candiduria, whereas ICU admission, broad-spectrum antibiotics, and DM were common risk factors for candidemia. The demographic features, predisposing factors, clinical conditions, and patient outcomes with *C. auris *infection are shown in Table 3.

**Table 3:** Demographic characteristics, predisposing factors, clinical features and outcome of patients with C. auris infection. SRC tip: self-retaining catheter; ALD: alcoholic liver disease; AKI: acute kidney injury; CKD: chronic kidney disease; IHD: ischemic heart disease; MV: mechanical ventilation; DM: diabetes mellitus; HTN: Hypertension; FLZ: fluconazole; LAMA: leave against medical advice; Y: yes; N: no; DAMA: discharge against medical advice; spp: species

	1	2	3	4	5	6	7	8	9
Clinical specimen	Urine	Urine	SRC tip	Urine	Urine	Urine	Blood	Blood	High vaginal swab
Clinical presentation	ALD, variceal bleeding	Sepsis,	Sepsis	CKD, HTN	ALD, pneumothorax	DM, pneumonia	Septic shock, AKI, IHD, DM	Sepsis with septic shock, multiorgan dysfunction, Dengue shock syndrome	24.4 weeks of amenorrhea, PV leakage
Surgical intervention	N	N	N	N	N	N	N	N	N
Indwelling medical device	Foley’s catheter	MV, Foley’s catheter	MV, Foley’s catheter	Foley’s catheter	MV, Foley’s catheter, central line	MV, Foley’’s catheter	MV, Foley’s catheter, central line	MV, Foley’s catheter	N
Antifungal prophylaxis/treatment	Y (FLZ)	Y (FLZ)	Y (FLZ)	Y (FLZ)	Y (FLZ)	N	Y (FLZ)	N	N
Bacterial co-infection from other source	Y (Pseudomonas spp.)	Y (Klebsiella spp.)	Y (Klebsiella spp.)	Y (Klebsiella spp., Pseudomonas spp).	Y (Klebsiella spp., E. coli)	Y (Klebsiella spp.)	Y (Klebsiella spp., Acinetobacter spp. )	N	N
Broad spectrum antibiotics	Y	Y	Y	Y	Y	Y	Y	Y	Y
Previous hospitalization	Y	Y	Y	Y	Y	Y	Y	Y	Y
Predisposing factors	HTN, liver disease	CKD, DM	CKD, DM	CKD, HTN	Liver disease	DM	DM	AKI	N
Length of hospitalization	9 days	5 days	5days	14 days	6 days	10 days	12 days	02 days	5 days
Outcome	LAMA	Death	Death	LAMA	Death	Death	Death	Death	DAMA

## Discussion

*C. auris*, an emerging multidrug-resistant yeast, is capable of causing life-threatening infections in hospitalized patients. Since its first identification in 2009, *C. auris* has spread across the world [8]. The ability to form a biofilm, the capability of long-term colonization, along with resistance to commonly used disinfectants make both control and elimination of *C. auris* difficult in a hospital setting [5]. As this pathogenic yeast has reduced susceptibility to azoles, it often results in therapeutic failure and eventually with high mortality rates [ 9]. *C. auris* is a unique cryptic isolate from the NAC spp., as it has every potential of disseminating in healthcare setups more robustly compared to *C. albicans* and other species of Candida. Infections due to *C. auris* are usually hard to treat as this pathogen is resistant to antifungals of routine use. In contrast to *C. albicans* and other pathogenic species of Candida, *C. auris* remains unidentifiable by conventional methods used for speciation of Candida spp [5, 8, 9].

Here, we analysed 147 Candida species isolated from different clinical specimens for a period of 10 months. With consistent to recent national and international studies, a predominance of NAC species was noted in the current study. NAC species were isolated from 96 (65.3%) clinical specimens. *C. tropicalis* was the commonest NAC species It was isolated from a total of 47 clinical specimens. Previous Indian studies have reported *C. tropicalis* as the predominant species [1].

The exact prevalence of *C. auris* from India is largely unknown as most diagnostic microbiological services rely mostly on conventional methods for identifying Candida species. In this study, *C. auris* produced pink-colored colonies on HiChrome Candida differential agar, the chromogenic media used for species distribution of clinically important Candida species pink-colored colonies are produced by several NAC species, and hence it is difficult to identify *C. auris* solely on the basis of colony color and morphology on chromogenic media. The problem with the identification of *C. auris* becomes more grave because the automated systems are available at few centers whilst advanced methods like MALDI-TOF are seldom performed at most of the clinical microbiology laboratories for Candida, hence it is possible that *C. auris* may be underreported from India [10]. 

VITEK 2 has recently added *C. auris* to its database. By using this commercial system, we could identify a total of nine *C. auris* from various clinical specimens. These isolates were confirmed by performing MALDI-TOF. Identification of *C. auris* from cultures is difficult given the morphologic similarities to other yeasts, its slow growth, and the low culture sensitivity when using standard agars and temperatures [11]. Therefore, this study underscores the urgent need to shift Candida identification from conventional to advanced automated systems.

Out of nine *C. auris*, five were from urine specimens. In recent years, *C. auris* has been increasingly isolated from cases of urinary tract infections (UTI) globally [12]. As per published literature, the treatment of patients with *C. auris* UTI is highly variable and ranges from apparently no treatment to combination antifungal therapy for prolonged durations [13]. Although the Centers for Disease Prevention and Control (CDC) recommendations are against treating non-invasive *C. auris* infections, published reports implicate treatment of *C. auris* infections, regardless of isolation site. Ignoring and not treating *C. auris* UTI may increase the risk of disseminated infections that are very difficult to treat and often associated high morbidity and mortality [13].

In this study, one isolate of *C. auris* was from a high vaginal swab collected from a 26 years old female with 26 weeks of amenorrhea who was admitted for a complaint of PV leakage for 13 days. The patient was diagnosed with premature rupture of membrane (PROM). As the patient opted for discharge against medical advice (DAMA) after two days of admission, the further outcome of *C. auris* vaginal colonization could not be assessed. An earlier study has documented colonization of the birth canal as a source of colonization in preterm and extremely low birth weight neonates [14].

Disseminated *C. auris* infections are of concern and among major health issues in patients admitted to critical care areas as they often lead to more severe outcomes either alone or in combination with other comorbidities. In this study, out of 16 isolates of Candida from blood cultures, two isolates (12.5%) were identified as *Candida auris*, which were associated with severe systemic illnesses, including acute kidney injury (AKI), ischemic heart disease (IHD), diabetes mellitus (DM), and sepsis with multiorgan dysfunction, including dengue shock syndrome. Since the first reported isolation of *C. auris* in India in 2013, the cases of BSI due to this pathogen have incrementally reported from various Indian hospitals [15]. *C. auris* is reported to be fourth or fifth among the various species of Candida isolated from BSI in most hospitals of this country [15].

As shown in Table 3, all patients were on broad-spectrum antibiotics, whereas six patients received prophylactic/therapeutic fluconazole. This highlights the importance of the implementation of an antimicrobial stewardship program (AMSP) along with the judicious use of fluconazole. A total of eight (88.9%) of these patients had a history of multiple hospitalizations and were admitted to ICU. Prolonged hospital stays, with a mean of 7.56 days significantly increase the risk of *Candida auris* infections due to extended exposure to healthcare environments and invasive procedures. Chowdhary et al. (2017) highlighted that stays longer than seven days elevate infection risk [16]. Risk factors also included comorbid conditions like CKD, HTN, DM, and medical devices, including catheters. These patients also had concomitant bacterial infections. A similar observation was made by Al-Rashdi et al (2021) [17].

The mortality rate of *C. auris* infection is high and varies between 30% and 50% [18]. In this study, a total of six patients succumbed to their conditions, which included hypertension (HTN), sepsis, multi-organ failure, and acute liver disease (ALD), whereas two patients left against medical advice (LAMA), and one patient opted for DAMA. However, out of six patients who succumbed, three showed isolation of *C. auris* from urine culture whereas one *C. auris* isolated was from SRC tip. The exact reasons for fatality can’t be ascertained as either *Candida auris* as well as underlying condition may be the cause. To the best of our knowledge, this is the first study to report the isolation of *C. auris* not from this hospital but from this specific geographical area of rural Maharashtra, providing essential baseline data for future research and public health strategies. This pioneering work enhances our understanding of the diagnostic challenges associated with identifying *C. auris* among various Candida species, which is crucial for the early recognition and treatment of infections. Additionally, the findings will aid healthcare providers in implementing effective infection prevention and control measures to combat the growing threat posed by *C. auris*.

Limitation

A limitation of this study was the time needed to confirm the *C. auris* species from a MALDI-TOF-equipped laboratory situated in another city, which delayed timely communication with the concerned consultant. Only nine *Candida auris *were studied, indicating a low prevalence rate in the research. However, a longer duration and multicentric study will be necessary to validate statistically the risk factors associated with *Candida auris* infection.

## Conclusions

*Candida auris* is emerging as a healthcare concern with a high rate of treatment failure and mortality. As *C. auris* has every quality to disseminate in a healthcare setup, it is very important to be vigilant against this dreadful yeast pathogen. In this study, *C. auris* was most predominantly isolated from urine specimens. Since invasive infections due to *C. auris* have notably high mortality rates, urinary tract infections should not be ignored as at times they may progress to disseminated infection, especially in patients with comorbidities. Our study highlights the importance of automated systems for identifying *C. auris*. The prompt and accurate identification and specific antifungal drugs will play a crucial role in treating and implementing enhanced infection prevention and control measures.
